# Prostaglandin E2 receptor 3 (EP3) signaling promotes migration of cervical cancer via urokinase-type plasminogen activator receptor (uPAR)

**DOI:** 10.1007/s00432-020-03272-0

**Published:** 2020-06-01

**Authors:** Yao Ye, Lin Peng, Aurelia Vattai, Eileen Deuster, Christina Kuhn, Christian Dannecker, Sven Mahner, Udo Jeschke, Viktoria von Schönfeldt, Helene H. Heidegger

**Affiliations:** 1 Department of Gynecology and Obstetrics, Xinhua Hospital, Shanghai Jiao Tong University School of Medicine, Shanghai, China; 2 Department of Obstetrics and Gynecology, University Hospital, Ludwig-Maximilians University of Munich, Campus Großhadern: Marchioninistraße 15, 81377 Munich, Germany; 3 Department of Obstetrics and Gynecology, University Hospital, University of Augsburg, Augsburg, Germany

**Keywords:** Cervical cancer, Prostaglandin E_2_ receptor 3 (EP3), Urokinase-type plasminogen activator receptor (uPAR), Plasminogen activator inhibitor type 1 (PAI-1)

## Abstract

**Purpose:**

Cervical cancer metastasis results in poor prognosis and increased mortality, which is not separated from inflammatory reactions accumulated by prostaglandin E2 (PGE2). As a specific G-protein coupled PGE2 receptor, EP3 is demonstrated as a negative prognosticator of cervical malignancy. Now, we aimed to investigate the pathological mechanism of EP3 in modulating cervical cancer carcinogenesis.

**Methods:**

Bioinformatics analysis was used to identify PAI-1 and uPAR correlations with EP3 expression, as well as the prognosis of cervical cancer patients. In vitro analyses were carried out to investigate the role of EP3 on cervical cancer proliferation and migration.

**Results:**

In vitro studies showed that sulprostone (an EP3 agonist) enhanced the proliferation and migration of cervical cancer cells, whereas silencing of EP3 inhibited their proliferation and migration. Furthermore, EP3 knockdown increased the expression of plasminogen activator inhibitor type 1 (PAI-1), urokinase-type plasminogen activator receptor (uPAR), and phosphorylated extracellular signal-regulated kinases 1/2 (p-ERK1/2), but decreased p53 expression. Bioinformatics analysis showed that both PAI-1 and uPAR were correlated with EP3 expression, as well as the prognosis of cervical cancer patients. The survival analysis further showed that uPAR overexpression (IRS≥2) was correlated with a lower overall survival rate of cervical cancer patients with advanced stages (FIGO III-IV).

**Conclusion:**

These results indicated that EP3 signaling pathway might facilitate the migration of cervical cancer cells through modulating uPAR expression. Therefore, EP3 and uPAR could represent novel therapeutic targets in the treatment of cervical cancer in advantaged stages.

**Electronic supplementary material:**

The online version of this article (10.1007/s00432-020-03272-0) contains supplementary material, which is available to authorized users.

## Introduction

Cervical cancer is the fourth most common cancer in women worldwide and approximately 510,000 new cases of women will be diagnosed in 2030 as today (Ginsburg et al. 2017). According to the cancer statistics of the United States in 2019, there were an estimated 13,170 cases and 4250 deaths from cervical cancer (Siegel et al. 2019). Approximately half of the cervical cancer patients die from metastasizing tumors globally (Wright and Kuhn 2012). The two main malignant epithelial cervical cancer types are the squamous cell carcinoma and the adenocarcinoma (Young and Clement 2002). The human papillomavirus (HPV) infection is the crucial risk factor for cervical cancer and is the primary cause of cervical cancer (Schiffman et al. 2011). Inflammation after HPV infection is a driving force that increases cervical cancer development (Deivendran et al. 2014). Cyclooxygenase-2 (COX-2) and prostaglandin E_2_ (PGE_2_) are well-known inflammatory factors and up-regulated synthesis of both has been identified in the cervical carcinoma (). As the rate-limiting enzyme of PGE_2_ synthesis, COX-2 is not only related to poor overall survival (OS) and poor disease-free survival (DFS) in cervical cancer patients, but also is associated with poor DFS in a chemo-radiation subgroup of cervical cancer patients (Huang et al. 2013).

The effects of PGE_2_ are mainly facilitated by four specific membrane-bound G-protein-coupled EP receptors (EP1-EP4) with various signaling pathways (Sokolowska et al. 2015). It is suggested that PGE_2_ regulates the function of cervical cancer cells mainly via cyclic adenosine monophosphate (cAMP) linked EP2/EP4 signaling pathway (). GW627368X (a highly selective EP4 antagonist) inhibits the proliferation and angiogenesis of cervical carcinoma by blocking EP4/epidermal growth factor receptor (EGFR) signaling pathway in cervical cancer cell lines (HeLa, SiHa and ME180) and suppresses the tumor size in xenograft mice model (Parida et al. 2016). Our latest publication demonstrated that high expression of EP3 is associated with poor prognosis in overall survival rates of cervical cancer patients in both squamous cell carcinoma and adenocarcinoma (Heidegger et al. 2017). EP3 is a unique PGE_2_ receptor, since the human EP3 gene consists of ten exons and nine introns, encoding at least eight distinct EP3 splice variants (Kotani et al. 1997). EP3 has been reported to mediate the carcinogenesis in numerous tumors with conflicting effects (Fujino et al. 2011; Hoshikawa et al. 2009; Kang et al. 2011; Kashiwagi et al. 2013; Ma et al. 2013; Shoji et al. 2004; Yamaki et al. 2004; Zhu et al. 2018). However, the molecular pathological mechanism of EP3 in cervical cancer development is still unknown.

Plasminogen activator contributes to proteolytic degradation and intercellular interaction damage during tumor metastasis. Plasminogen activator inhibitor type 1 (PAI-1) is the main inhibitor of the plasminogen activating system, which consists of urokinase-type plasminogen activator (uPA) and uPA receptor (uPAR) (Zorio et al. 2008). PGE_2_ combining with the EP1/EP3 receptor regulates the levels of PAI-1 in cardiac fibroblasts (Kassem et al. 2014). PAI-1 inhibits the activation of uPA and plays a crucial role in cancer invasion and metastasis by remodelling the extracellular matrix (ECM). PAI-1 enhances tumor cell proliferation by encouraging S-phase entry (Giacoia et al. 2014) and increases migration by binding uPA/uPAR complex (Andreasen et al. 2000). PAI-1/uPA/uPAR/low-density lipoprotein receptor-related protein (LRP)/integrin complexes are initiating an “adhesion–detachment–re-adhesion” cycle to promote tumor cell migration (Andreasen et al. 2000; Carter and Church 2009). Overexpressions of both PAI-1 (Hazelbag et al. 2004; Horn et al. 2002) and uPA (Fujishiro et al. 1994; Sugimura et al. 1992) are associated with poor prognosis in cervical cancer patients. However, Sato et al. proposed that lower levels of PAI-1 are produced in cervical cancer cells that distant from the basal membrane, especially in cervical cancer stem cells (Sato et al. 2016). These conflicting reports indicate the complex roles of PAI-1 in cervical carcinoma development, which requires further investigations. The uPAR protein in the serum (Jing et al. 2012) and uPAR mRNA in the specimen (Sasaki et al. 2014) are identified as new prognosticators of cervical cancers. uPAR can be cleaved into soluble uPAR, both full-length and cleaved uPAR are involved in cell signaling, proliferation, migration and invasion of tumor cells (Magnussen et al. 2017). However, the correlation between uPAR and overall survival of cervical cancer has not been clarified.

In the present study, we aimed to explore the functional roles of EP3 in the tumor genesis of cervical cancer, especially in the migration. In in vitro studies, we observed that EP3 silencing attenuated the proliferation and migration of cervical cancer cells and upregulated the expression of PAI-1 and uPAR. This was in accordance with the finding that EP3 was significantly correlated with PAI-1 and uPAR from publicly available databases. By immunohistochemistry, we demonstrated that high uPAR expression was associated with the poor prognosis of cervical cancer patients with advanced stages (FIGO III–IV). Our present study shed light on the critical role of EP3 and uPAR in regulating migration in cervical cancer in advantaged stages.

## Materials and methods

### Bioinformatics

The gene set enrichment analysis (GSEA) software was performed to calculate the corresponding signaling pathways associated with EP3 (https://www.software.broadinstitute.org/gsea/index.jsp). The cut-off criteria for GSEA were nominal *P* value < 0.05 and false discovery rate (FDR) < 0.25. TIMER database was applied to identify the correlation between EP3 and PAI-1 or uPAR (https://cistrome.shinyapps.io/timer/). Both of GSEA and TIMER databased are based on the cervical squamous cell carcinoma and endocervical adenocarcinoma (CESC) in the Cancer Genome Atlas (TCGA) dataset (https://www.cancer.gov). We analyzed the survival rate in groups with differently expressed PAI-1 and uPAR by screening out the relevant documents and clinical information related to CESC in GEPIA database (https://gepia.cancer-pku.cn/) and UALCAN database (https://ualcan.path.uab.edu/index.html), respectively.

### Cell lines and culture

HeLa (RRID:CVCL_0030), SiHa (RRID: CVCL_0032), C-33A (RRID: CVCL_1094) and CaSki (RRID: CVCL_1100) cells were obtained from the American Type Culture Collection (ATCC) and were cultured in RPMI-1640 medium (Gibco, USA) supplemented with 10% fetal bovine serum (FBS, Gibco, USA) without antibiotics or antimycotics. According to the American Type Culture Collection (ATCC), HeLa cells are categorized as cervical adenocarcinoma, SiHa cells are squamous cell carcinoma, CaSki cells are categorized as epidermoid carcinoma and C-33A cells are categorized as cervical carcinoma. All experiments were performed with mycoplasma-free cells. To investigate the effect of EP3 knockdown, cells were cultured in 96-well plates for the cell proliferation assay, 24-well plates for the wound healing assay and the enzyme-linked immunosorbent assay (ELISA), and 6-well plates for real-time polymerase chain reaction (RT-PCR) and western blotting.

### Real time-PCR (Taq Man)

Total RNA was obtained from cultured cells using a Rneasy Mini Kit (Qiagen, Hilden, Germany) and converted to cDNA with an MMLV Reverse Transcriptase First-Strand cDNA synthesis kit (epicenter, Madison, USA) as instructed by the protocol. The total EP3 mRNA levels were subjected to RT-PCR using two different primers (Applied Biosystems, EP3 Primer I, Nr. Hs00168755_m1, exon boundary 1–2; EP3 Primer II, Nr. Hs00988369_m1, exon boundary 4–5). 20 μl reaction mixture containing 1 μl TaqMan^®^ Gene Expression Assay 20 ×, 10 μl TaqMan^®^ Fast Universal PCR Master Mix 2 ×, 1 μl cDNA template and 8 μl RNase-free water were prepared per probe on an Optical Fast 96-well plate and covered by an optical adhesive film. PCR assays were run by utilizing Applied Biosystems 7500 Fast Real-time PCR system. The amplification conditions were 20 s at 95 °C; 40 cycles of 95 °C for 3 s and of 60 °C for 30 s. β-actin (Nr. Hs99999903_m1) was used as an endogenous control and the comparative CT method was applied for calculation.

### EP3 silencing

Cervical cancer cells (HeLa, SiHa and C-33A) were seeded in six-well plates in 2 ml of RPMI-1640 medium to achieve 40–60% confluence after 24 h. 1.2 µl of EP3 siRNA or the negative control siRNA and 4 µl of Lipofectamine RNAiMAX (Invitrogen, California, USA) were first diluted in 200 µl Opti-MEM (Gibco, California, USA) medium separately. Then we combined and added the corresponding complex into each well, mixed gently, and incubated at 37 °C in 5% CO_2_ for 48 h. The knockdown efficiency was assessed by RT-PCR.

### Cell proliferation assay

HeLa, SiHa and C-33A cells were seeded into 96-well plates and siRNA-mediated EP3 knockdown was conducted with the siRNA-Lipofectamine RNAiMAX mixture on day two. Cell proliferation was analyzed with a 5-bromo-2′-deoxy-uridine (BrdU) labeling and detection kit (Roche Diagnostics GmbH, Mannheim, Germany) according to the manufacturer’s instructions. Cells were incubated with BrdU (20 µl/well) for 24 h, and then fixed with fixing solution for 30 min. After adding anti-BrdU-POD working solution (100 µl/well), BrdU incorporation into the cellular DNA was measured by an ELISA technique. The optical density (OD) was examined at 450 nm using Elx800 universal Microplate Reader. At least six replicates were performed with each cell line. 100 nM of PGE_2_ and L-798,106 were incubated with HeLa, SiHa and C-33A cells and the dimethyl sulfoxide (DMSO, 0.5%) served as a vehicle control. The BrdU assay was performed as describe above.

### Wound healing assay

HeLa and SiHa cells were cultured in 24-well plates, starved overnight and on day two siRNA-mediated EP3 knockdown was treated for 48 h. On day three, the central fields of confluent monolayers were scratched with 200 µl pipette tips to make artificial wound gaps. Then each well was rinsed with phosphate-buffered saline (PBS) and was then added fresh RPMI1640 containing 1% FBS. Cell migration was monitored by photographing with an inverse phase contrast microscope (Leica Dmi1, Leica, Wetzlar, Germany) for 0 h and 24 h. Photos of cells migration area were analyzed with software Image J (https://imagej.nih.gov/ij/). Scratch area was measured at 0 h and 24 h by image J, and the cell migration area = scratch area at 0 h—scratch area at 24 h.

### Western blotting

Cell lysates were extracted from cervical cancer cells with radioimmunoprecipitation assay buffer (RIPA, Sigma-Aldrich, R0278-50ML). 20 µg of cell lysates for western blotting were first separated in 10% sodium dodecyl sulfate–polyacrylamide gel electrophoresis and then transferred to a polyvinylidene fluoride membrane (Bio-Rad, USA). The membrane was blocked in 4% skim milk powder and then incubated with the primary antibodies for 16 h at room temperature. Different primary antibodies were used as follows: rabbit polyclonal anti-EP3 antibody (Abcam, ab94496, 1:500), mouse polyclonal anti-ERK1/2 antibody (Abcam, ab224313, 1:200), rabbit polyclonal anti-p-ERK1/2 antibody (Abcam, ab47339, 1:500), mouse monoclonal anti-p53 antibody (Santa Cruz, OD-1, 1:500) and rabbit polyclonal anti-uPAR antibody (Abcam, ab218106, 1:300). β-actin was used as a housekeeping gene and mouse monoclonal anti-β-actin antibody was diluted as 1:1000 (Sigma, A5441). Afterwards, the membrane was incubated with the goat-anti-rabbit/-mouse secondary antibody conjugated with alkaline phosphatase (1:1000 dilution, Jackson Immuno Research, UK), and detected with 5-bromo-4-chloro-3′-indolylphosphate/nitro-blue tetrazolium (BCIP/NBT)-chromogen substrate solution (Promega). Western blots were scanned and quantified using the GelScan V6.0 1D Analysis Software (SERVA, Electrophoresis GmbH, Heidelberg, Germany). The blots were repeated at least three times.

### PAI-1 ELISA

Both HeLa and SiHa cells were cultured in 24-well plates and EP3 knockdown was conducted utilizing the siRNA-Lipofectamine RNAiMAX mixture on day two. After 48 h, the supernatants of both cell lines were harvested. The levels of PAI-1 in the supernatants were measured with a commercially available enzyme-linked immunosorbent assay (ELISA) kit (R&D system, DSE100, Minneapolis, MN, USA). A standard curve of PAI-1 was obtained for each assay and results were converted into ng/ml.

### Patient samples

We analyzed paraffin-embedded cervical cancer samples from 250 patients having undergone surgeries for cervical cancer in the Department of Obstetrics and Gynecology in the Ludwig Maximilians University of Munich, Germany between 1993 and 2002. This study was approved by the ethical committee of the Medical Faculty, Ludwig Maximilian University of Munich (approval number: 259-16). The written informed consent was obtained from each patient and all methods were performed in accordance with the relevant guidelines and regulations. Staging and grading were assessed by two gynecological pathologists according to the criteria of FIGO and WHO. Follow-up data were received from the Munich Cancer Registry (Munich Tumour Center, Munich, Germany). Samples and clinical information were anonymized and encoded for statistical workup. All clinical information was blinded from the authors during experimental analysis.

Detailed clinic characteristics of these cervical cancer patients are summarized in supplementary Table 1, which includes age, follow-up months, stages, grading, histology and survival months. The outcome was assessed by patients’ overall survival (OS). OS is defined as the time from diagnosis to the death or to the date of the last follow-up. 76% (190/250) of the cohort survived over 235 months and 19.6% (49/250) of the cohort died. The information of the rest 4.4% (11/250) of the cervical cancer patients is missing.

**Table 1:**
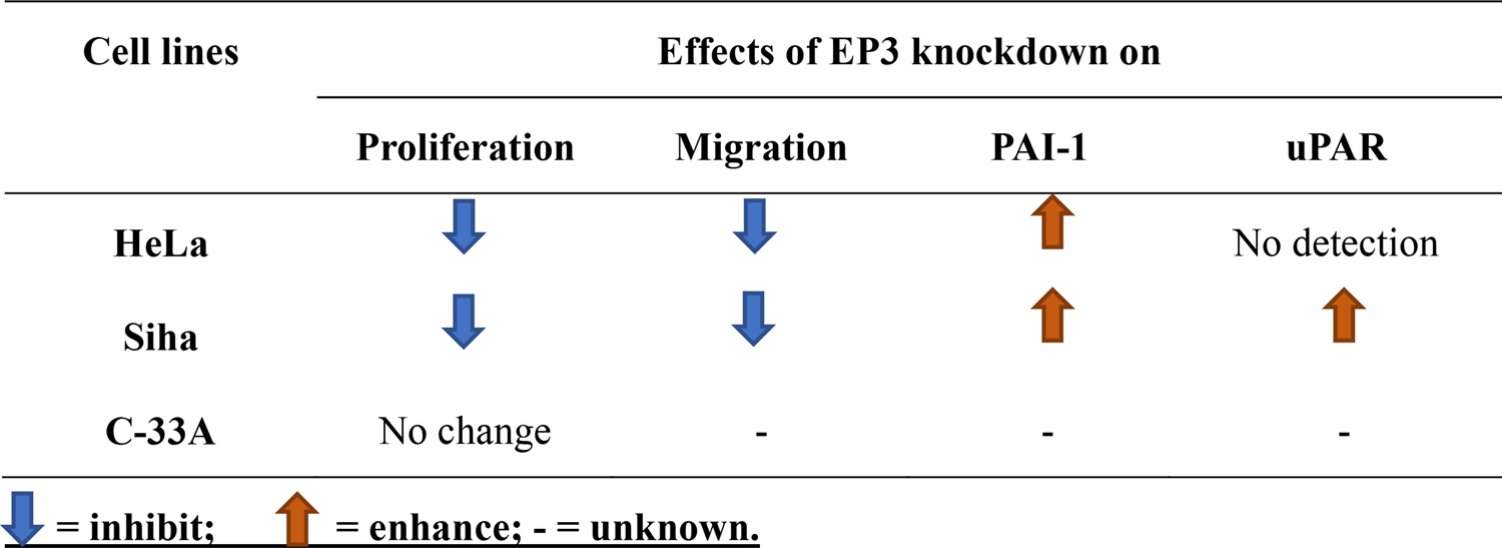
Effects of EP3 knockdown in HeLa, Siha and C-33A cervical cancer cell lines

### Immunohistochemistry

Paraffin-embedded slides (3 µm-thick) were dewaxed in xylol and washed in 100% ethanol, and then were incubated in methanol with 3% H_2_O_2_ and rehydrated in a descending alcohol series. Slides were heated in a pressure cooker using sodium citrate buffer (pH = 6.0), containing 0.1 M citric acid and 0.1 M sodium citrate in distilled water. After cooling and washing in PBS, all slides were incubated with a blocking solution [Reagent 1, Zytochem-Plus HRP-Polymer-Kit (mouse/rabbit)] for 30 min to avoid non-specific binding of the primary antibodies. The slides were incubated with rabbit polyclonal anti-uPAR antibody (Abcam, ab218106, 1:300 dilution) for 16 h at 4 °C. After washing, the secondary antibodies/complexes of HRP-polymer (Zytochem-Plus HRP Polymer-kit, Zytomed, Berlin, Germany) were applied. uPAR immunostaining was visualized with the substrate and the chromogen-3, 3′-diaminobenzidine (DAB; Dako, Hamburg, Germany) after 3 min. All slides were analyzed under the microscope by two independent observers using a Leitz (Wetzlar, Germany) photomicroscope. For the light microscopy analysis, a semi-quantitative IRS score was calculated via the multiplication of optical staining intensity and the percentage range of positive stained cells (Remmele and Stegner 1987).

Metastatic colon carcinoma was used as a positive and negative control for the immunohistochemical staining of uPAR. Positive cells showed a brownish color and the negative control, as well as unstained cells, appeared blue (Worbs et al. 2007).

### Statistical analysis

All data were analyzed with SPSS Statistics 24 software (IBM Corporation, Armonk, NY, USA) and are expressed as the mean ± standard deviation (SD). Mann–Whitney* U* test was applied for evaluating the proliferation rate and cell migration area. Wilcoxon test was performed for the evaluation of PAI-1 expression levels and the band intensities of p-ERK1/2, ERK1/2, p53 and uPAR. Spearman’s rank correlation analysis was adopted to evaluate the correlation between two monotonic, nonlinear variables. The ROC curve was drawn to identify an appropriate cut-off value which can maximize the sum of sensitivity and specificity. Survival time was compared using Kaplan–Meier (long-rank) test method. We also applied a Cox-regression model for multivariate analyses. *P*-values < 0.05 were regarded as statistically significant.

## Results

### Associated EP3 signaling pathways were upregulated in cancer

**Fig. 1:**
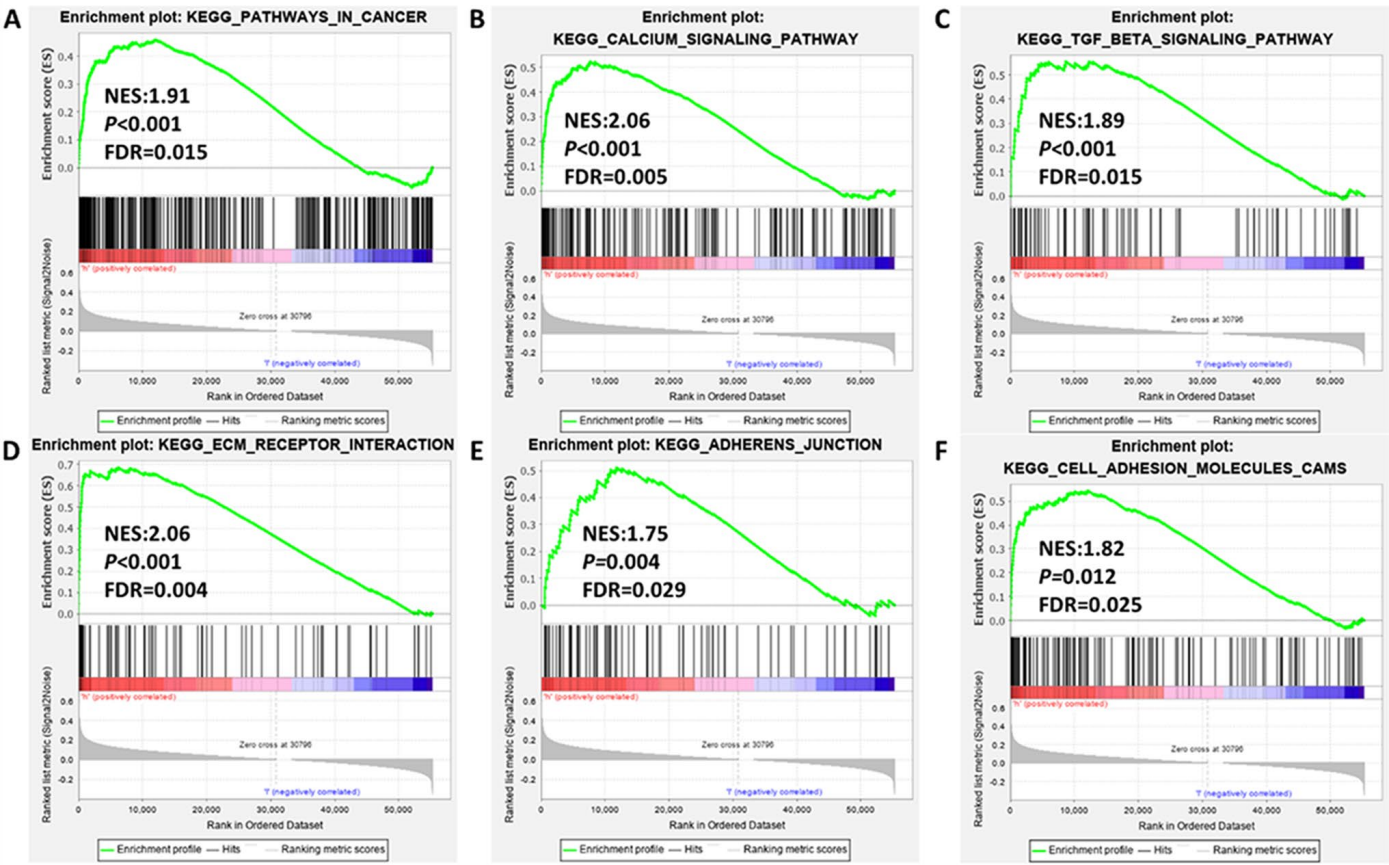
EP3 is associated with KEGG signaling pathways of cancer (**a**), calcium signaling (**b**), transforming growth factor-β (TGF-β) (**c**), ECM receptor interaction (**d**), adheren junction (**e**) and cell adhesion molecules (CAMs) (**f**) in carcinogenesis. KEGG pathway gene sets in EP3 high versus low samples were obtained from The Cancer Genome Atlas (TCGA) dataset with the gene set enrichment analysis (GSEA) software (https://www.software.broadinstitute.org/gsea/index.jsp). Normalized enrichment score (NES), nominal *P* value and false discovery rate (FDR) are shown in each plot. The cut-off criteria for GSEA were nominal *P* value < 0.05 and false discovery rate (FDR) < 0.25

Our latest publication observed that enhanced expression of EP3 (IRS ≥ 2) is correlated with a poor prognosis in the OS of 250 cervical cancer patients after a 20-year follow-up analysis (Heidegger et al. 2017). Additionally, increased EP3 expression is associated with higher tumor status, higher the International Federation of Gynecology and Obstetrics (FIGO)-classification, as well as with poorer survival (Heidegger et al. 2017). Based on this publication, we aimed to investigate the pathological mechanism of EP3 in the carcinogenesis of cervical cancer. First, we analyzed the relationship between EP3 expression and KEGG pathway gene sets with GSEA software (https://www.software.broadinstitute.org/gsea/index.jsp). Pathways in cancer, calcium signaling and transforming growth factor-β (TGF-β) signaling were significantly enriched (Fig. 1a–c), so were ECM receptor interaction, adheren junction and cell adhesion molecules (CAMs) signalings (Fig. 1d–f). This indicated that EP3 might be involved in the carcinogenesis, especially in tumor adhesion, migration and metastasis.

### Knockdown of EP3 inhibits the proliferation and migration of HeLa and SiHa cells

**Fig. 2:**
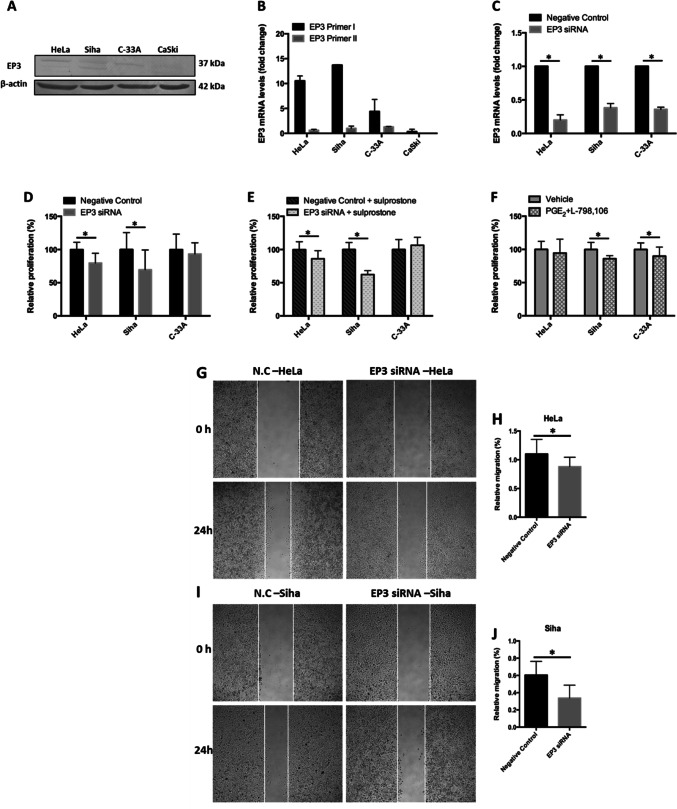
EP3 knockdown inhibits the proliferation and migration of cervical cancer cells. **a** The expression of EP3 is higher in HeLa, SiHa and C-33A than CaSki cells in the protein level by western blots. **b** The expression of EP3 is higher in HeLa, SiHa and C-33A than CaSki cells in the mRNA level detected by primer I with RT-PCR. **c** The downregulated expression of EP3 mRNA is shown in HeLa, SiHa and C-33A detected by RT-PCR (*P < 0.05). **d** BrdU assay suggests the proliferation rate of HeLa and SiHa is decreased by EP3 knockdown compared to the negative control after 48 h. **e** The proliferation rate of HeLa and SiHa is inhibited followed by stimulation of 100 nM sulprostone and EP3 siRNA compared to the negative control after 48 h (*P < 0.05). **f** The proliferation rate of SiHa and C-33A is decreased by 100 nM of PGE_2_ and L-798,106 compared to the vehicle control after 48 h (0.5% (v/v) DMSO, *P < 0.05). **g** Representative photographs show the migration of HeLa cells into the wounded area treated with the EP3 siRNA and the negative control after 24 h. **h** We observed that the relative migration rate of HeLa cells is suppressed in the EP3 siRNA group compared to the negative control (*P < 0.05). **i** Representative pictures represent the migration of SiHa cells into the wounded area followed by incubating EP3 siRNA and the non-targeting control for 24 h. **j** The relative migration rate of SiHa cells is inhibited in the EP3 siRNA group compared to the non-targeting control (*P < 0.05). Bar graphs represent mean ± SD (*n* = 6). *P < 0.05 is considered as significantly different after comparison between the EP3 siRNA and the negative control (N.C)

Next, we investigated the effect of EP3 knockdown on the proliferation and migration of cervical cancer cells with in vitro cell culture. The EP3 expression levels in HeLa, SiHa, C-33A and CaSki cervical cancer cell lines were determined by western blotting and real-time polymerase chain reaction (RT-PCR) analyses. The protein expression of EP3 was higher in HeLa, SiHa and C-33A cells than CaSki cells detected by western blots (Fig. 2a). With the EP3 primer I, the expression of EP3 in the mRNA level (Fig. 2b) showed the similar result as western blots. With the EP3 primer II, the mRNA expression of EP3 was detected only in HeLa, SiHa and C-33A cells, and was not as high as with the EP3 primer I (Fig. 2b). Therefore, we used HeLa, SiHa and C-33A as cervical cancer models and the EP3 primer I for RT-PCR detection after the depletion of EP3 mRNA with siRNA. The EP3 mRNA level was downregulated by 80% in HeLa cells, 62% in SiHa cells and 64% in C-33A cells compared to the negative control, respectively (each *P* < 0.05, Fig. 2c).

EP3 knockdown decreased the proliferation rate by 20.3% in HeLa cells (*P* = 0.028, Fig. 2D) and by 30.5% in SiHa cells (*P* = 0.036, Fig. 2d) compared to the relative negative control group after 48 h’ incubation. Since sulprostone (an EP1/EP3 agonist) can enhance the proliferation of HeLa cells (*P* = 0.028 at the concentration of 1, 10 and 100 nM, supplementary Fig. 1a), we tested the effect of EP3 siRNA on the proliferation of sulprostone-induced cervical cancer cells. As shown in Fig. 2e, co-incubation of EP3 siRNA and 100 nM sulprostone reduced the proliferation rate by 13.9% in HeLa cells (*P* = 0.043) and by 37.7% in SiHa cells (*P* = 0.028) compared with the non-targeting siRNA with 100 nM sulprostone after 48 h’ incubation. By contrast, the proliferation rate of C-33A cells was neither significantly altered by EP3 siRNA (*P* = 0.33, Fig. 2dD), nor by the combination of 100 nM sulprostone and EP3 siRNA (*P* = 0.075, Fig. 2e).

The inhibitory effect was exhibited when incubating 100 nM of PGE_2_ and L-798,106 (a specific EP3 antagonist) with SiHa and C-33A cells, although the effect was not as obvious as the effect of EP3 siRNA. The combination of PGE_2_ and L-798,106 suppressed the proliferation rate by 14.0% in SiHa cells (*P* = 0.028, Fig. 2f) and by 10.0% in C-33A cells (*P* = 0.046, Fig. 2f) compared to the vehicle (0.05% DMSO) after incubating for 48 h, respectively.

To identify whether EP3 participates the migration of cervical cancer cells, we performed wound healing assay. Our pre-test showed that 100 nM sulprostone promoted the migration rate of HeLa cells by 13.6% for 24 h (*P* = 0.015, supplementary Fig. 1b, c). In comparison, EP3 knockdown inhibited the migration rate by 20.0% in HeLa cells (*P* = 0.016, Fig. 2g, h) and by 44.2% in SiHa cells (*P* = 0.006, Fig. 2i, j) compared with the negative control. To wrap it up, downregulation of EP3 inhibited the proliferation and migration of HeLa and SiHa cells, while had no effect on C-33A cells. The impacts of EP3 knockdown on the proliferation, migration and expression of PAI-1/uPAR in HeLa, Siha and C-33A cells were summarized in Table 1.

### EP3 is correlated with PAI-1 and uPAR in cervical cancer tissues

**Fig. 3:**
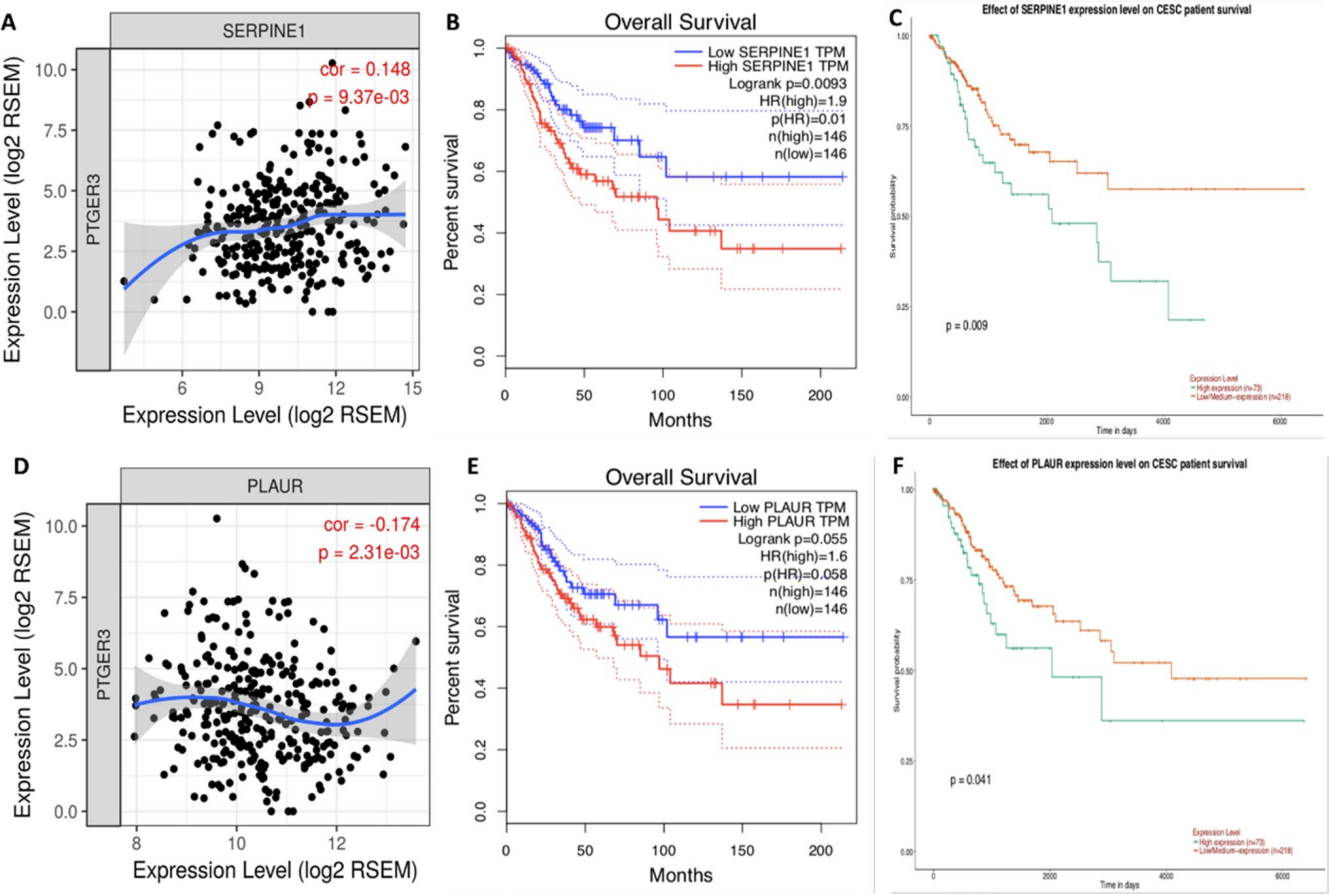
EP3 is correlated with PAI-1 and uPAR in cervical cancer. **a, d** TIMER database was applied to identify the correlation between EP3 and PAI-1 or uPAR, which is based on the CESC (cervical squamous cell carcinoma and endocervical adenocarcinoma) in the Cancer Genome Atlas (TCGA) dataset (https://www.cancer.gov). **b, c** PAI-1 is associated with poor overall survival (OS) of cervical cancer patients both in GEPIA database (https://gepia.cancer-pku.cn/) and UALCAN database (https://ualcan.path.uab.edu/index.html). **e, f** The association of uPAR with poor prognosis of cervical cancer patients is significant in UALCAN database but not in GEPIA database

Migration and invasion are responsible for the majority of patients death from solid tumors in advanced stages (Paul et al. 2017), and both of PAI-1 and uPA are involved in the migration of cervical carcinoma (Fujishiro et al. 1994; Hazelbag et al. 2004; Horn et al. 2002; Sugimura et al. 1992). TIMER database was applied to identify the correlation between EP3 and PAI-1 or uPAR. The result showed that EP3 was positively correlated with PAI-1 (*r* = 0.148, *P* = 9.37 × 10^–3^, Fig. 3a) and negatively correlated with uPAR (*r* = − 0.174, *P* = 2.31 × 10^–3^, Fig. 3d). With the GEPIA and UALCAN databases we observed that the OS of the low PAI-1 expression group was higher than that of the high PAI-1 expression group in the long run (*P* = 0.0093 in GEPIA, Fig. 3b; *P* = 0.009 in UALCAN, Fig. 3c). The OS was not significantly different in the low and high uPAR expression groups in GEPIA database (*P* = 0.055, Fig. 3e), whereas the OS of the low uPAR expression group was increased than that of the high uPAR expression group in UALCAN (*P* = 0.041, Fig. 3f). Therefore, we examined the expression of PAI-1 and uPAR in HeLa and SiHa cells after knocking down EP3 and then testified uPAR expression in our 250 cervical cancer specimens because of the diverse results of two databases.

### Knockdown of EP3 increases the expression of PAI-1 and uPAR

**Fig. 4:**
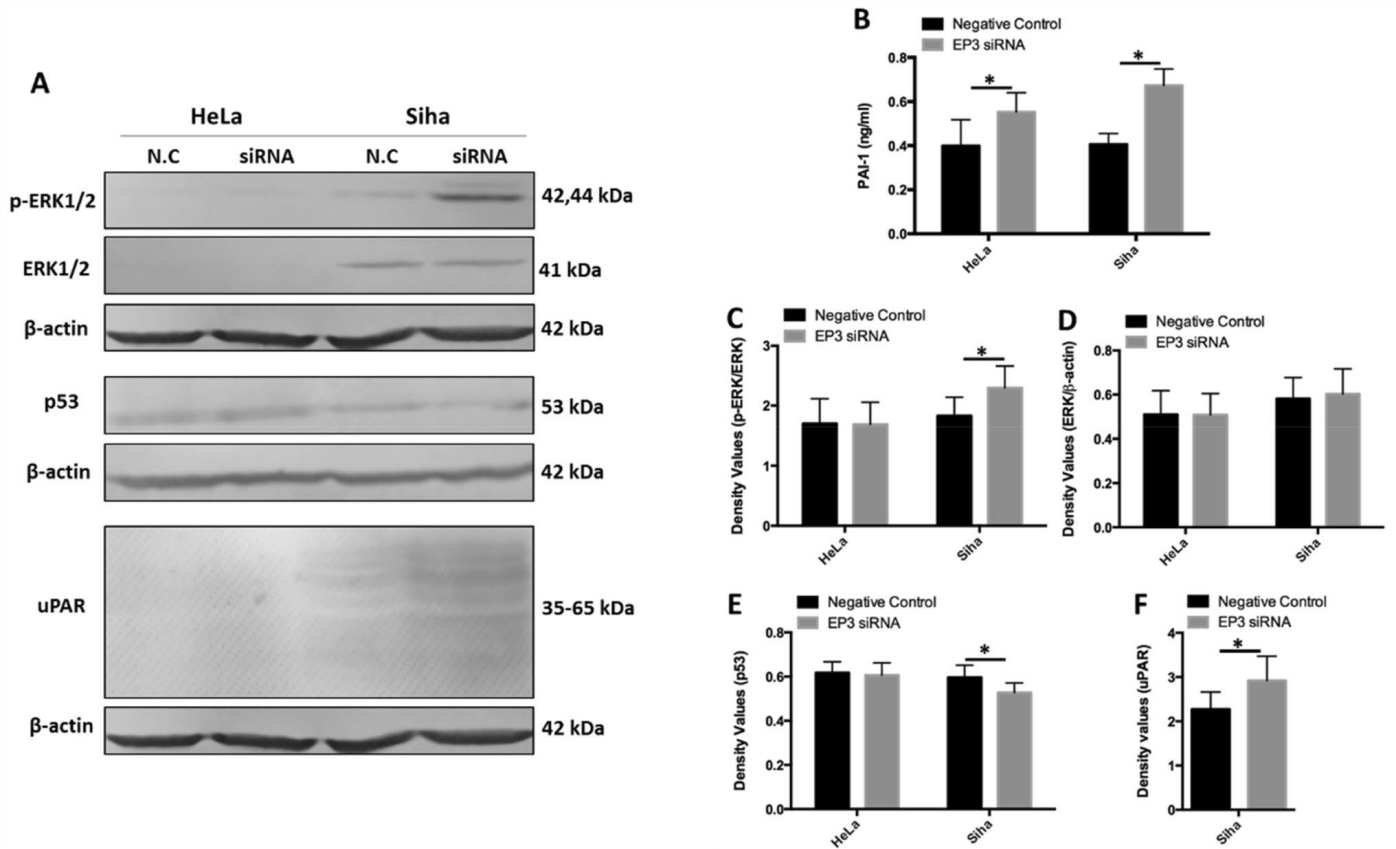
Expression of plasminogen activator inhibitor type 1 (PAI-1) and urokinase-type plasminogen activator receptor (uPAR) is influenced by silencing EP3 gene. **a** Western blotting analysis shows the expression of phosphorylated extracellular signal-regulated kinases (p-ERK1/2), extracellular signal-regulated kinases (ERK1/2), p53 and uPAR in HeLa and SiHa cells following treatment with EP3 siRNA and the negative control (N.C) for 48 h. β-actin was used as a loading control and all the data was normalized to the β-actin band signals. **b** PAI-1 levels in the supernatants of HeLa and SiHa cells are enhanced after silencing EP3 compared with the negative control for 48 h by ELISA (*P < 0.05, *n* = 6). **c** The histogram illustrates the expression of p-ERK1/2 is increased after silencing EP3 gene for 48 h in SiHa cells (*P < 0.05). **d** The histogram presents the expression of ERK1/2 is not altered by EP3 siRNA in HeLa and SiHa cells (*P* > 0.05). **e** The histogram illustrates the expression of p53 is inhibited after downregulation of EP3 compared with the negative control for 48 h in SiHa cells (*P < 0.05). **f** The histogram shows the expression of uPAR is stimulated after EP3 knockdown compared with the negative control for 48 h in SiHa cells (*P < 0.05). Statistically significant differences (*P* < 0.05) between EP3 siRNA group and the negative control group are marked with an *. All western blots data are shown as mean ± SD (*n* = 3). Full-length blots are shown in Supplementary Fig. 2

Knockdown of EP3 promoted the production of PAI-1 in the supernatants of both HeLa and SiHa cells. Downregulation of EP3 enhanced the production of PAI-1 by 38.7% in the supernatants of HeLa cells compared to the negative control (0.55 ± 0.09 vs 0.40 ± 0.12 ng/ml, *P* = 0.003, Fig. 4b). The same trend was observed in SiHa cells, downregulation of EP3 increased the production of PAI-1 in the supernatants by 66.1% compared to the negative control (0.67 ± 0.07 vs 0.41 ± 0.05 ng/ml, *P* = 0.003, Fig. 4b). Both phosphorylated extracellular signal-regulated kinases 1/2 (p-ERK1/2) and p53 are the upstream regulators of PAI-1 gene transcription (Samarakoon et al. 2013; Wilkins-Port et al. 2007), hence we also analyzed the expression of p-ERK1/2 and p53 by western blots. The molecular weights of p-ERK1/2 are 44 and 42 kDa. In SiHa cells, EP3 blockade increased the expression of p-ERK1/2 by 25.5% (*P* = 0.028, Fig. 4a, c) while did not change the expression of total ERK 1/2 (*P* = 0.753, Fig. 4a, d) compared to the negative control group after incubating EP3 siRNA for 48 h. Silencing EP3 decreased the expression of p53 by 7.4% in SiHa cells (*P* = 0.011, Fig. 4a, d). Additionally, the expression of uPAR was also analyzed by western blotting and the molecular weight of uPAR is between 35–65 kDa. EP3 knockdown improved the expression of uPAR by 28.6% in SiHa cells (*P* = 0.027, Fig. 4a, e). No alteration of p-ERK1/2, ERK1/2 and p53 was observed in HeLa cells while the expression of uPAR was not able to be detected in HeLa cells (Fig. 4a).

### Expression of uPAR in cervical cancer tissues

**Fig. 5:**
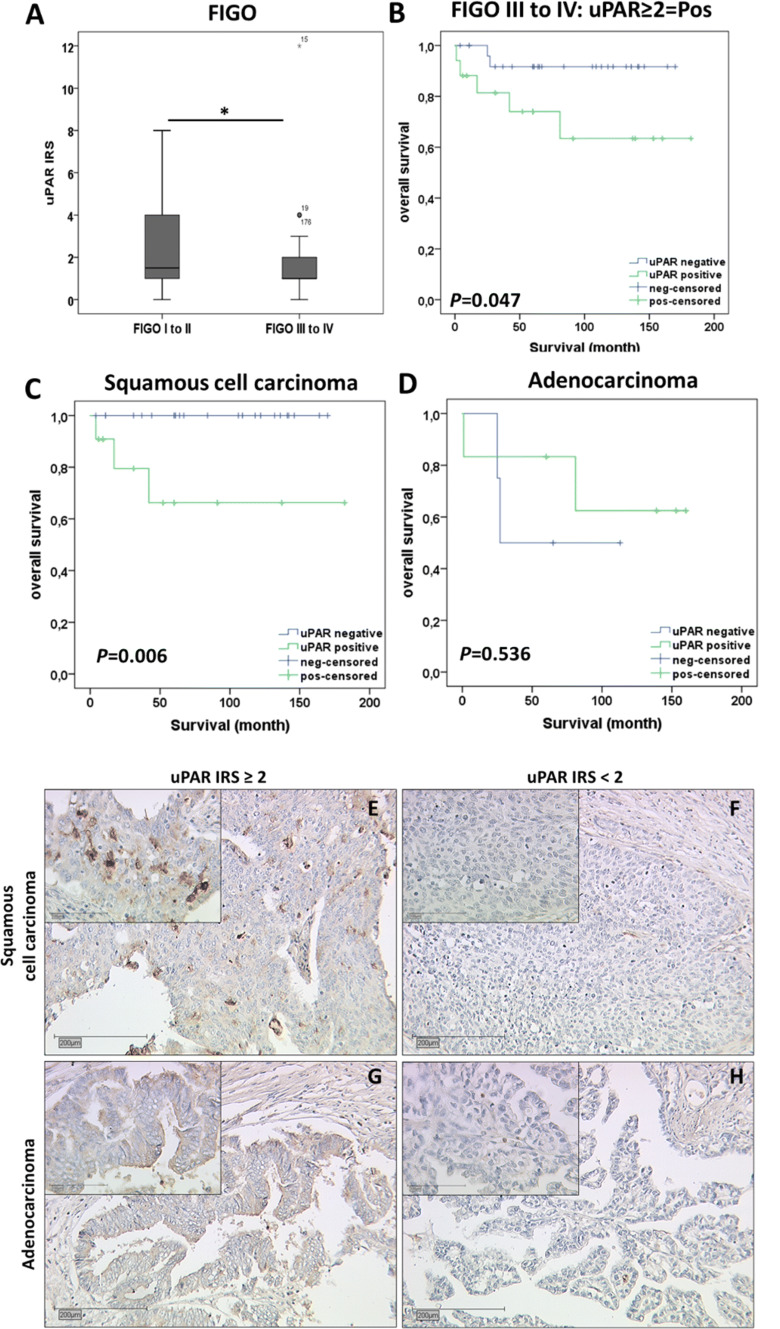
The expression of urokinase-type plasminogen activator receptor (uPAR) in cervical cancer patients. **a** Boxplot shows uPAR staining in cervical cancer patients with FIGO I and II is higher than in cases with FIGO III and IV (*P* = 0.046). **b** High uPAR expression (IRS ≥ 2) is associated with a shorter overall survival (OS) in advanced cervical cancer patients (FIGO III/IV) (*P* = 0.047). **c** High uPAR expression (IRS ≥ 2) is associated with a shorter OS of advanced patients in cervical squamous cell carcinoma (*P* = 0.006). **d** uPAR survival function of cervical adenocarcinoma in patients with FIGO stages III/IV (*P* = 0.536). **e** Representative photomicrographs of uPAR staining in cervical squamous cell carcinoma (FIGO IIIB) with the IRS score of 3. **f** Representative photomicrographs of uPAR staining in cervical squamous cell carcinoma (FIGO IIIB) with the IRS score of 0. **g** Representative photos of uPAR immunohistochemical staining in adenocarcinoma (FIGO IIIA) with the IRS score of 4. **h** Representative photos of uPAR immunohistochemical staining in adenocarcinoma (FIGO IVB) with the IRS score of 0. The scale bars in the outer pictures equal 200 µm (× 10 magnification) and the scale bars in the inserts equal 100 µm (× 50 magnification). *FIGO* the International Federation of Gynecology and Obstetrics

Finally, we analyzed uPAR expression in the same group of 250 cervical cancer patients as we previously conducted (Heidegger et al. 2017) and examined the correlation of uPAR expression with clinical-pathological parameters and several cervical cancer biomarkers. uPAR staining was observed in the cytoplasm of 93.6% (234/250) of cervical cancer tissue samples, and the median IRS for cytoplasmic uPAR expression was 2.05. Although EP3 was negatively correlated with uPAR in the TIMER database, there was no significant correlation between uPAR and EP3 expression in our cervical cancer specimens (*P* = 0.822, Table 2). However, a significant negative correlation was shown between uPAR expression and FIGO status (spearman’s rank correlation Rho = − 0.165; *P* = 0.012), suggesting the weaker uPAR staining was correlated with a higher FIGO stage (Table 1). Additionally, decreased uPAR staining was observed in cervical cancer cases with higher FIGO stages (*P* = 0.046, Fig. 5a). A total of 44.8% (112/250) of cervical cancer patients with FIGO stages I/II had a median IRS of 2.17 compared to 17.6% of patients (44/250) with FIGO stages III/IV and with a median IRS of 1.52 (Fig. 5a).

**Table 2:** Correlation analysis of uPAR and variables

Variables	*P* value	Correlation coefficient
Histology	0.247	0.076
pT	0.117	− 0.103
pN	0.017*	− 0.156
pM	0.308	− 0.067
Grading	0.397	0.056
FIGO	0.012*	− 0.165
EP3	0.822	− 0.015
E6	0.836	0.014
p16	0.05*	− 0.130
Wild-type p53	0.118	− 0.103
Mutant p53	0.082	− 0.114
MDM2	0.938	− 0.005
galectin-3	0.002*	− 0.202
GPER	0.52	− 0.042
H3K9ac	0.121	− 0.102
H3K4me3	0.041*	0.134

Bold numbers represent significant correlations

*pT* tumor stage, *pN* lymph node stage, *pM* distant metastasis stage, *FIGO* the International Federation of Gynecology and Obstetrics, *EP3* prostaglandin E_2_ receptor 3, *MDM2* MDM2 proto-oncogene, *GPER* G-protein-coupled estrogen receptor, *H3K9ac* histone H3 acetyl K9, *H3K4me3* histone H3 tri methyl K4

The cut off value of IRS 2 was obtained from receiver operator curve (ROC) analysis. We observed that uPAR positivity (IRS ≥ 2) in general was not related to OS in our non-stratified patient samples (*P* = 0.48). However, when patients had been stratified according to FIGO stage, the high expression of uPAR was correlated with poor prognosis in OS of cervical cancer patients with FIGO stages III/IV as shown in the Kaplan–Meier curve (*P* = 0.047, Fig. 5b). Among all the 44 advanced cervical cancer patients (FIGO III/IV), 34 cases with squamous cell carcinoma had a median IRS of 1.12 and 10 cases with adenocarcinoma had a median IRS of 2.9, which showed no significant difference between these two histological subtypes (*P* = 0.09). The subsequent survival analysis of the two main histological subtypes suggested a significant negative correlation of uPAR with OS in squamous cell carcinoma (*P* = 0.006, Fig. 5c), but not in cervix adenocarcinoma (*P* = 0.536, Fig. 5d). The representative cytoplasmic expression of uPAR in the squamous cell carcinoma and adenocarcinoma were shown in Fig. 5, and metastatic colon carcinoma tissues were applied as negative and positive controls (Supplementary Fig. 3A, B). It indicated that immunopositivity of uPAR was predictive for OS in cervical cancer patients of advanced stage (FIGO III/IV), especially among cases with squamous cell carcinoma. In advanced cervical cancer patients (FIGO III/IV), uPAR was nearly a promising prognosticator for advanced cervical cancer patient OS (*P* = 0.067, Table 3) tested by multivariate Cox regression analysis.

**Table 3:** Cox regression of clinical-pathological variables regarding overall survival in cervical cancer patients with FIGO III/IV (*n* = 44)

Variable	Significance	Hazard ratio of exp (B)	Lower 95% CI of exp (B)	Upper 95% CI exp (B)
uPAR IRS	0.067	8.332	0.863	80.425
Histology	0.222	5.182	0.370	72.505
pT	0.231	2.056	0.632	6.687
pN	0.987	4,553,661.9	0.000	–
pM	0.314	0.193	0.008	4.734
Grading	0.255	3.664	0.391	34.350
FIGO	0.962	0.974	0.331	2.865
Age	0.588	1.031	0.922	1.153

*IRS* Immunoreactive score, *pN* lymph node stage, *pT* tumor stage, *pM* distant metastasis stage, *FIGO* the International Federation of Gynecology and Obstetrics

Moreover, we detected a significant positive correlation of uPAR with histone H3 tri methyl K4 (H3K4me3, *P* = 0.041, Rho = 0.134) and a significant negative correlation with pN (*P* = 0.017, Rho = − 0.156), p16 (*P* = 0.05, Rho = − 0.13) and galectin-3 (*P* = 0.002, Rho = − 0.202) in 250 cervical cancer tissues (Table 1). The percentage of uPAR positive staining cells was negatively associated with the intensity of wild-type p53 staining in the cytoplasm (*P* = 0.011, Rho = − 0.184, data not shown), although no correlation between uPAR and wild-type nuclear p53 (*P* = 0.118) or between uPAR and mutant p53 in the nucleus (*P* = 0.082).

## Discussion

Our latest study demonstrated that high expression of EP3 (IRS ≥ 2) is associated with poor prognosis in the OS rate of 250 cervical cancer patients in both squamous cell carcinoma and adenocarcinoma (Heidegger et al. 2017). EP3 can increase the migration of HCA-7 human colon cancer cells through the activation of phosphatidylinositol 3-kinase (PI3K) and the phosphorylation of ERK1/2 signaling pathway (Fujino et al. 2011). In accordance to those findings, we found that sulprostone (an EP1/EP3 agonist) induced the proliferation and migration of HeLa cells, while silencing EP3 reduced the proliferation and migration of HeLa and SiHa cells. In contrast to colon cancer cells, EP3 silenced SiHa cells showed elevated expression of phosphorylated-ERK1/2. The latter was in accordance with a study demonstrating that activation of EP3 signaling reduced ERK phosphorylation in rat cerebellar astrocytes (Paniagua-Herranz et al. 2017).

With bioinformatics, signaling pathways of ECM receptor interaction, adheren junction and cell adhesion molecules were enriched when EP3 was upregulated in cancer microenvironment. Additionally, we observed that EP3 was positively associated with PAI-1 in cervical malignancy, and PAI-1 was correlated with the OS of cervical cancer patients in both UALCAN and GEPIA databases. Studies also proved that PAI-1 is an independent prognosticator in cervical cancer (Hazelbag et al. 2004; Horn et al. 2002). Therefore, we deduced that EP3 and PAI-1 are involved in the tumor migration of cervical tumor. PGE_2_ can increase mRNA and protein levels of PAI-1 by binding with EP1/EP3 receptor in rat ventricular fibroblasts, contributing to elevated fibrin deposition in aortic stenosis (Kassem et al. 2014). However, Sato et al. suggested that TM5275 (a small molecular inhibitor of PAI-1) can increase the collagenase activity of SiHa and CaSki cells (Sato et al. 2016), implying that lower expression of PAI-1 benefits the ECM degration and cervical cancer migration. The latter study was in accordance with our study that silencing EP3 increased the production of PAI-1 and decreased the migration in HeLa and SiHa cells. Conflicting effects of PAI-1 on migration might due to the different distances of cervical cancer cells from basal membrane (Sato et al. 2016).

In comparison, EP3 expression was negatively associated with uPAR expression in CESC. The correlation of uPAR with OS of cervical cancer patients was significant in UALCAN but not GEPIA. Sasaki et al. testified that overexpression of uPAR mRNA is related to a shorter DFS of cervical cancer patients, however, the immunohistochemical staining of uPAR was not very intense (Sasaki et al. 2014). In the present study, we detected only those patients with an uPAR expression (IRS ≥ 2) showed a poor OS in the subgroup of advanced stage (FIGO III/IV) cases. The negative correlation of uPAR with OS of patients was significant in squamous cell carcinoma but not in cervix adenocarcinoma, which could be due to the smaller number of patients with adenocarcinoma (*n* = 10) or different pathological molecular mechanisms in squamous cell carcinoma and adenocarcinoma. This result agreed with multivariate Cox regression analysis, indicating that with a large scale of specimens, uPAR could be a promising prognosticator for OS of advanced cervical cancer patients.

**Fig. 6:**
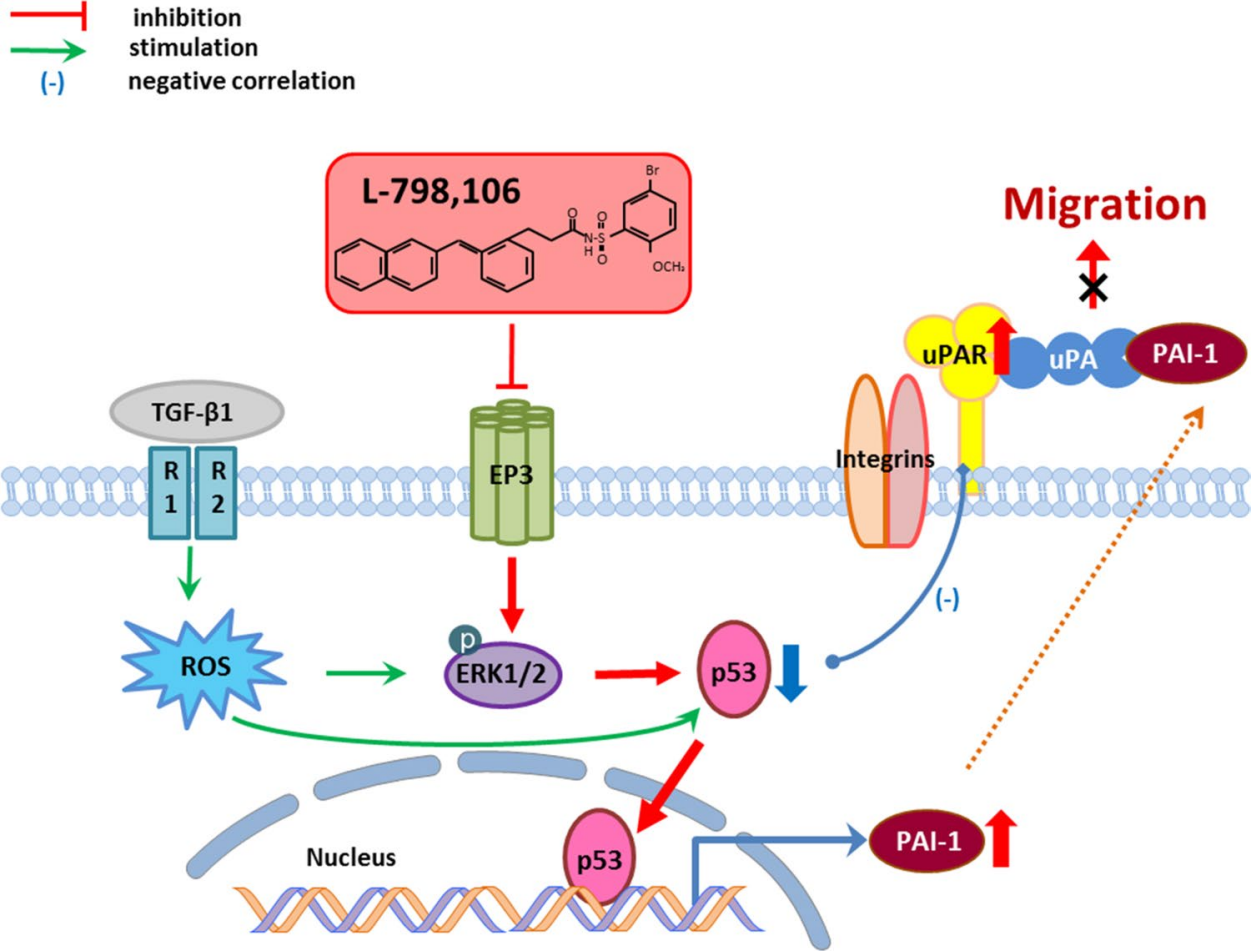
Hypothetic schema of EP3 signaling in the migration of human cervical cancer cells. Inhibiting EP3 signaling contributes to phosphorylation of extracellular signal-regulated kinases (p-ERK1/2) and translocation of p53 from the cytoplasm to the nucleus, resulting in an increased transcription of PAI-1. High expression of PAI-1 reduces uPAR cleavage (Magnussen et al. 2017), thus leading to decreased migration of cervical cancer cells. The EP3 signaling pathway is similar to the one that transforming growth factor-β1 (TGF-β1) induces PAI-1 gene expression via the rapid generation of reactive oxygen species (ROS), phosphorylation of ERK1/2 and the mobilization of p53 signaling (Samarakoon et al. 2013; Wilkins-Port et al. 2007). In addition, cytoplasmic p53 is decreased in the cervical cancer cells with high expression of uPAR, which is correlated with poor prognosis in overall survival rates of cervical cancer patients with advanced FIGO stages (III/IV). Therefore, we believed that EP3 signaling regulates the migration of cervical cancer cells through plasminogen activator inhibitor type 1 (PAI-1) and urokinase-type plasminogen activator receptor (uPAR)

Magnussen et al. (2017) proved that high production of PAI-1 can reduce uPAR cleavage to inhibit the migration of oral squamous cell carcinoma (OSCC) and the cleaved soluble uPAR is responsible for promoting the migration of OSCC cells (Magnussen et al. 2017). Jing et al. also suggested that the soluble uPAR in serum is a prognosis marker as well as a tumor biomarker for clinical diagnosis and treatment of cervical cancer (Jing et al. 2012). The in vitro studies showed knockdown of EP3 increased expression of uPAR and PAI-1 in SiHa cells. Therefore, we deduced that the upregulated secretion of PAI-1 decreased uPAR cleavage in EP3 knockdown SiHa cells causing less soluble uPAR in the ECM and leaving more uPAR on the membrane, and these might contribute to decreased migration of SiHa cells (Fig. 6).

Interestingly, TGF-β signaling pathway was found to be significantly enriched when EP3 gene was upregulated in the CESC. Many studies illustrate the gene transcription of PAI-1 is regulated by TGF-β1 through various signalling pathways, one of which is through phosphorylation of ERK1/2 (Wilkins-Port et al. 2007). The cross-talks among reactive oxygen species (ROS), tumor suppressor p53, and upstream stimulatory factor proteins 1/2 (USF1/2) are necessary for TGF-β1 inducing PAI-1 transcription (Freytag et al. 2009; Samarakoon et al. 2013). This gave a hint that EP3 signaling possibly modulates PAI-1 gene transcription through similar signalling pathways as TGF-β1. We proved that EP3 blockade increased the expression of PAI-1 and p-ERK1/2 and decreased the expression of p53 in SiHa cells. Furthermore, decreased expression of wild-type p53 in the cytoplasm of cervical cancer tissues was correlated to increased expression of uPAR. This in vivo finding was in good agreement with the previous report that HPV E6 oncoproteins induce rapid degradation of tumor suppressor protein p53 to prevent the host cell from inducing apoptosis (DeFilippis et al. 2003). It implied that p53 might translocate into the nucleus from the cytoplasm to induce PAI-1 transcription. However, this deduction concerning p53 translocation should be explored in further studies.

Our group previously found other biomarkers of cervical cancer, such as p16 (Stiasny et al. 2017), MDM2 (Stiasny et al. 2017), galectin-3 (Stiasny et al. 2017), H3K9ac (Beyer et al. 2017) and H3K4me3 (Beyer et al. 2017). Therefore, we also analyzed the correlation of uPAR with these biomarkers in the same cervical cancer patients. First, we found that a negative correlation of uPAR with both galectin-3 and p16 in cervical cancer patients. Stiasny et al. showed that galectin-3 expression was correlated with a shorter survival time in cervical cancer patients expressing no or very low p16 (Stiasny et al. 2017). In hepatocellular carcinoma cells, galectin-3 silencing attenuated uPAR expression and inhibited the proliferation, migration and invasion (Zheng et al. 2014). This study was also in line with our detections that low expression of uPAR was correlated with longer survival time in cervical cancer patients with advanced stage. Additionally, the positive correlation of H3K4me3 and uPAR expression in our study was in accordance with the finding that H3K4me3 is related to poor prognosis in cervical cancer patients and is an independent marker of relapse-free survival (Beyer et al. 2017). Although EP3 seems not to correlate with the expression of the direct HPV marker protein E6 (*P* = 0.192, data not shown) or with the indirect protein p16 (*P* = 0.267, data not shown), there is a link between uPAR and p16 (*P* = 0.05, Table 2). This link was already found in migrating keratinocytes. Migration seems to be stimulated by a combined upregulation of both p16INK4a and an activated uPAR signaling (Darbro et al. 2005).

The upregulated expression of p-ERK1/2 was observed in SiHa cells while the expression of ERK1/2 was too low to draw any conclusion in HeLa cells. HeLa cells are categorized as adenocarcinoma and SiHa cells are squamous cell carcinoma according to the ATCC. The different pathological molecular mechanisms in cancer development should be investigated between squamous cell carcinoma and adenocarcinoma in the future. Another limitation of this investigation is that it is a retrospective study, which analyzed the data of patients who had undergone surgeries in one single hospital from 1993 to 2002. A multi-centre prospective study should be carried out for further research, as well as the xenograft mice experiments.

## Conclusions

Taken all results together, EP3 might facilitate the migration of cervical cancer cells through modulating the production of PAI-1 and uPAR in advantaged stages of cervical malignancy. The high production of PAI-1 might due to the phosphorylation of ERK1/2 and translocation of p53 from the cytoplasm into the nucleus after sliencing EP3. The high expression of PAI-1 inhibits the cleavage of uPAR (Magnussen et al. 2017), contributing to inhibited migration of cervical cancer cells (Fig. 6). EP3 and uPAR might represent novel therapeutic targets for cervical cancer and specific antagonists or inhibitors of EP3 and uPAR could be promising therapeutic treatments for cervical cancer.

## Electronic supplementary material

Below is the link to the electronic supplementary material.

Supplementary file1 (DOCX 5577 kb)

## Abbreviations

CESC: Cervical squamous cell carcinoma and endocervical adenocarcinoma
HPV: Human Papillomavirus
OS: Overall survival
DFS: Disease-free survival
PGE_2_: Prostaglandin E_2_
EP3: Prostaglandin E_2_ receptor 3
uPAR: Urokinase-type plasminogen activator receptor
PAI-1: Plasminogen activator inhibitor type 1
ECM: Extracellular matrix
ERK1/2: Extracellular signal-regulated kinases 1/2
p-ERK1/2: Phosphorylated Extracellular signal-regulated Kinases 1/2
TGF-β1: Transforming Growth Factor-β1
