# Supplementary material for: Prostaglandin E2 receptor 3 (EP3) signaling promotes migration of cervical cancer via urokinase-type plasminogen activator receptor (uPAR)
Source: J Cancer Res Clin Oncol. 2020 Jun 1;146(9):2189–203. doi: 10.1007/s00432-020-03272-0 (PMC7382663; doi:10.1007/s00432-020-03272-0)
Supplement: Supplementary file 1 — Supplementary file1 (DOCX 5577 kb) [file 432_2020_3272_MOESM1_ESM.docx]

**Supplementary Figure Legends and Tables**

**Supplementary Figure 1.** Sulprostone increases the proliferation and migration of HeLa cells. (*a*) BrdU assay shows that the proliferation rate of HeLa cells is elevated by 1, 10 and 100 nM of sulprostone (**P*<0.05). Results are normalized to cell viability of the control group (0.5% (v/v) DMSO). (*b*) We observed that the migration rate of HeLa cells is increased in the group treated with 100 nM sulprostone compared to the vehicle control (0.5% (v/v) DMSO) by wound healing assay after 24 hours (**P*<0.05). (*c*) Representative images show the migration of HeLa cells into the wounded area in the control group and in the group treated with 100 nM sulprostone. Bar graphs represent mean ± SD (n=6). **P*<0.05 is considered as significantly different after comparison between the stimulation group and the control group.

**
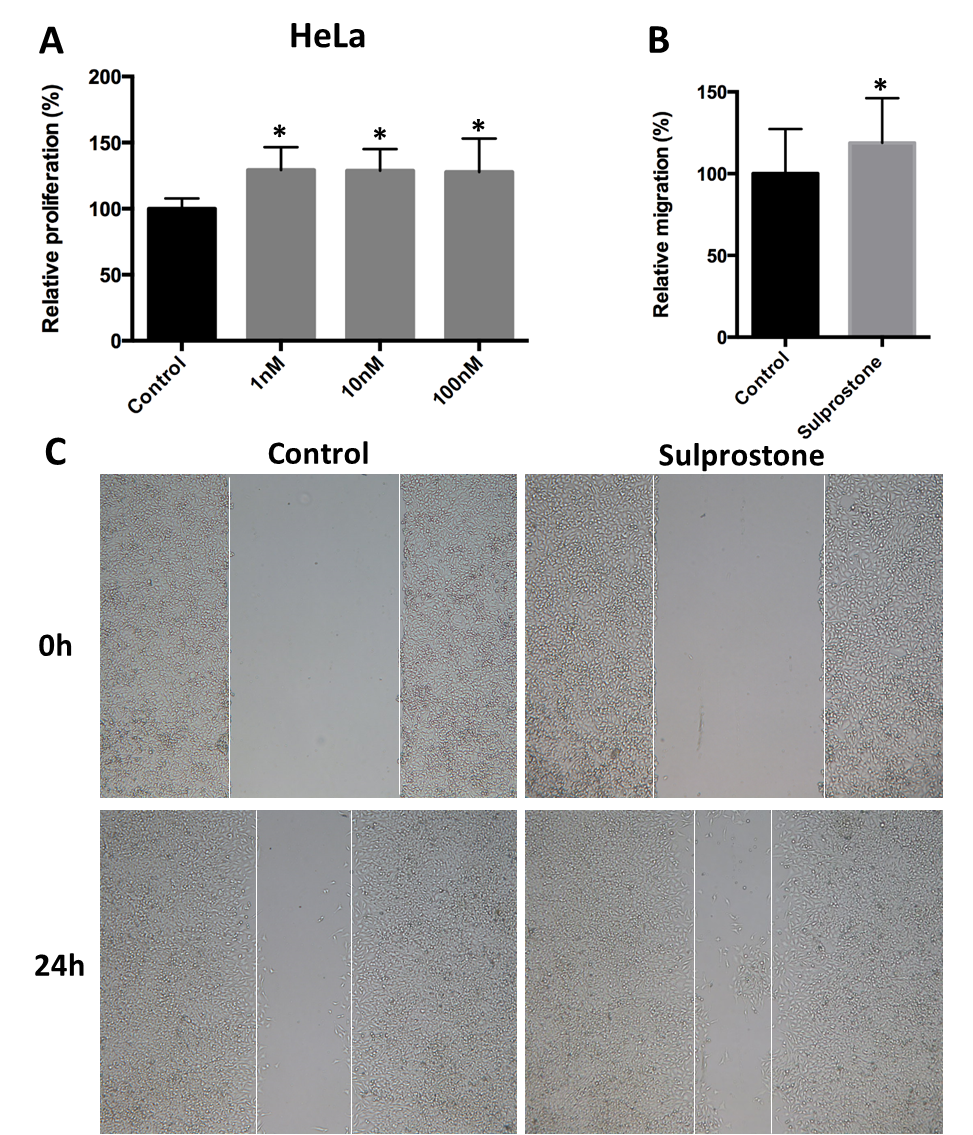
**

**Supplementary Figure 2.** Full-length blots of prostaglandin E2 receptor 3 (*a*) and the corresponding β-actin (*b*); p53 (*c*) and the corresoonding β-actin (*d*); phosphorylated extracellular signal-regulated kinases (p-ERK1/2) (*e*), extracellular signal-regulated kinases 1/2 (ERK1/2) (*f*), urokinase-type plasminogen activator receptor (uPAR) (*g*) and the corresponding β-actin (*h*).

**
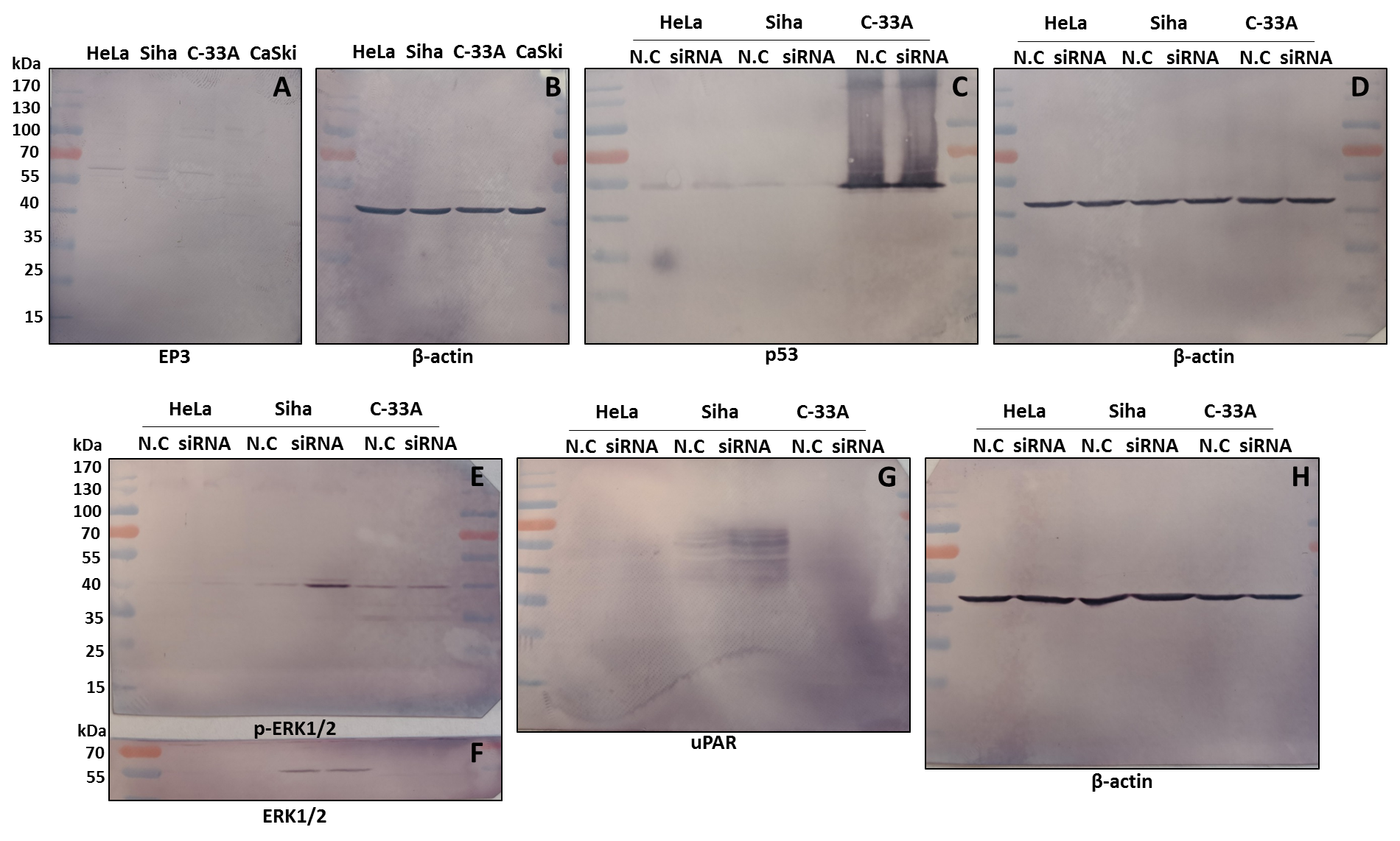
**

**Supplementary Figure 3.** Positive and negative controls of urokinase-type plasminogen activator receptor (uPAR) staining. We used the metastatic colon carcinoma for positive (*a*) and negative (*b*) controls.

**
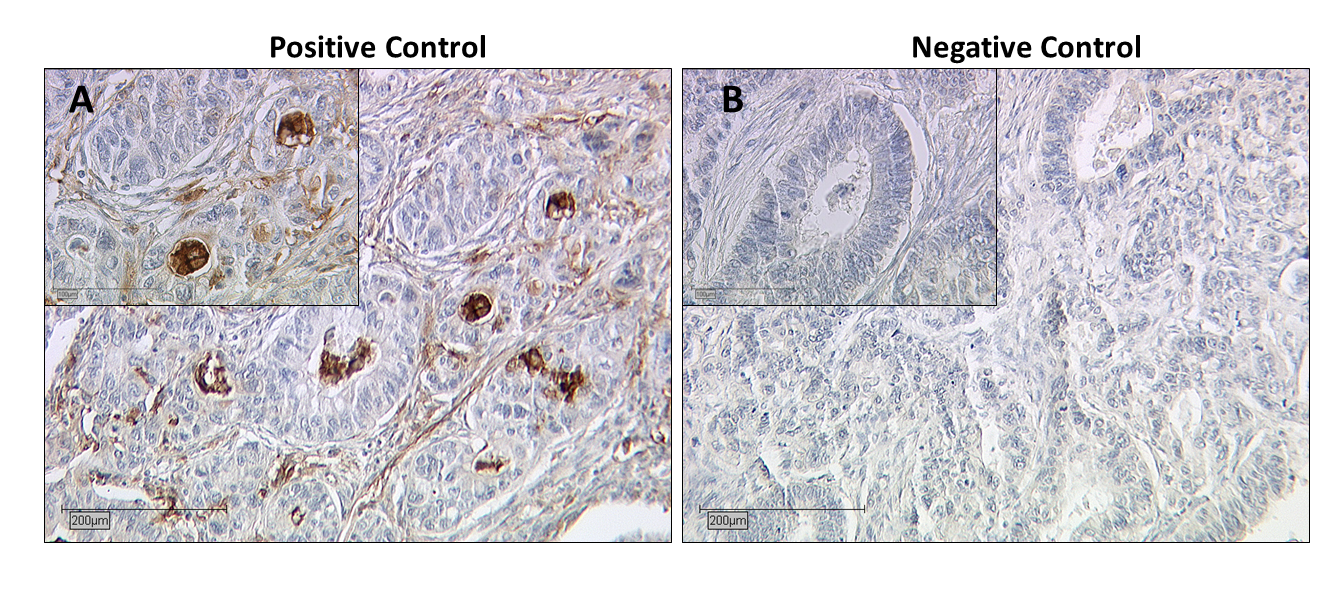
**

**Supplementary Table 1. Clinical-pathological variables of the patients included in the study**

| **Clinical characteristics** | **No./ Total No.** | **%** |
| --- | --- | --- |
| **Age** (Median) [years] | 40.5 |  |
| **Follow-up** (Median) [months] | 126.5 |  |
| **No. of Positive Nodes** |  |  |
| 0 | 151/250 | 60.4 |
| ≥1 | 97/250 | 38.8 |
| Not available | 2/250 | 0.8 |
| **FIGO** |  |  |
| I and II | 112/250 | 44.8 |
| III and IV | 44/250 | 17.6 |
| Not available | 94/250 | 37.6 |
| **Tumor Grade** |  |  |
| I | 21/250 | 8.4 |
| II | 143/250 | 57.2 |
| III | 78/250 | 31.2 |
| Not available | 8/250 | 3.2 |
| **Tumor Subtype** |  |  |
| Squamous | 202/250 | 80.8 |
| Adenocarcinoma | 48/250 | 19.2 |
| **Survival** (over 235 months) |  |  |
| Right censured | 190/250 | 76.0 |
| Died | 49/250 | 19.6 |
| Not available | 11/250 | 4.4 |

FIGO=the International Federation of Gynecology and Obstetrics
